# Method for automatic detection of defective ultrasound linear array transducers based on uniformity assessment of clinical images — A case study

**DOI:** 10.1002/acm2.12248

**Published:** 2018-01-11

**Authors:** Robert Lorentsson, Nasser Hosseini, Jan‐Olof Johansson, Wiebke Rosenberg, Benny Stenborg, Lars Gunnar Månsson, Magnus Båth

**Affiliations:** ^1^ Department of Medical Physics and Biomedical Engineering Sahlgrenska University Hospital Gothenburg Sweden; ^2^ Department of Radiation Physics Institute of Clinical Sciences at Sahlgrenska Academy University of Gothenburg Gothenburg Sweden

**Keywords:** transducer, ultrasound, uniformity

## Abstract

The purpose of the present study was to test an idea of and describe a concept of a novel method of detecting defects related to horizontal nonuniformities in ultrasound equipment. The method is based on the analysis of ultrasound images collected directly from the clinical workflow. In total over 31000 images from three ultrasound scanners from two vendors were collected retrospectively from a database. An algorithm was developed and applied to the images, 150 at a time, for detection of systematic dark regions in the superficial part of the images. The result was compared with electrical measurements (FirstCall) of the transducers, performed at times when the transducers were known to be defective. The algorithm made similar detection of horizontal nonuniformities for images acquired at different time points over long periods of time. The results showed good subjective visual agreement with the available electrical measurements of the defective transducers, indicating a potential use of clinical images for early and automatic detection of defective transducers, as a complement to quality control.

## 1 INTRODUCTION

There are many methods and recommendations on how to carry out performance tests and quality control on ultrasound systems.^1^, ^2^, ^3^, ^4^, ^5^, ^6^, ^7^, ^8^, ^9^, ^10^, ^11^ The transducer is a vital part of the system and handled daily. It is therefore subject to become defective. Not surprisingly, it has been shown that the incidence of defective transducers in clinical practice is high.^12^, ^13^, ^14^, ^15^, ^16^ As an example of a clinically relevant effect of defective transducers, the accuracy of Doppler measurements has been shown to be affected when several adjacent elements in the transducers are dysfunctional.^17^, ^18^

There are several methods for detecting defective transducers. The methods can be summarized as simple tests, tissue‐mimicking phantom tests and commercial electronic transducer tests. A simple and effective method is the “paper‐clip method”.^19^ A small piece of wire, such as a paper clip, is translated along the scan surface of the transducer in a small amount of gel, while the transducer is operating in air. By monitoring the display, scan line dropouts can be detected. Another simple method of detecting reduced sensitivity of transducer elements is to analyze an image with the transducer held in air.^7^, ^20^, ^21^ Another known technique for detection of defective transducers is to move a linear transducer along the arm while viewing the dynamic image; it is easy for a human eye to detect vertical streaks in the image when the tissue is moving while the streaks are not. This method, that uses several image frames, is based on the fact that every frame is slightly darker below the defective part of the transducer, since the ability to send and receive echoes is affected regardless if the failure is caused by dead elements, cable failure or delamination in the transducer. In a single image it may be difficult to discriminate these streaks from other details/structures in the image, but by using information from several image frames — where the image background varies but the streaks remain constant — the possibility for detection increases.

Visual inspection of the image uniformity using a tissue‐mimicking phantom is used in quality assurance to detect both vertical and horizontal nonuniformities.^3^ King et al.^22^ compared different ways of detecting defective transducers by using the information in several images of a dynamic clip of a low‐cost phantom produced for this purpose.^23^ In their study, the median image of the dynamic clip was used in two ways for visual assessment and was compared with assessment of the dynamic clip. All three methods were developed for increased sensitivity for detecting subtle artifacts compared to static single‐frame B‐mode images. The recently released IEC Technical Specification^24^ also uses the median image of a dynamic clip of a phantom acquired while moving the transducer slowly normal to the scan plane. A quantification of the horizontal nonuniformity of the image brightness is obtained by calculating a column‐wise median of the pixel values in the superficial part of the median image.

Electronic transducer testers such as FirstCall aPerio (Sonora Medical Systems, Inc., Longmont, CO, USA) and ProbeHunter (BBS Medical AB, Stockholm, Sweden) are testers to which the transducer is connected. Pulses are sent element‐wise toward a target in water and the echoes are evaluated. The result from the test is comprehensive and typically contains, among other parameters, the sensitivity for all elements individually presented as bar graphs. If an element has zero sensitivity, the element is nonfunctional.

The existing methods described above for testing for defective transducers are associated with limitations. Firstly, they all require access to the equipment (or at least the transducer) and are time‐consuming. This requires personal resources for performing the tests and the equipment is furthermore unavailable for clinical use. Secondly, when checking for defects, the time from failure to detection could in worst case be up to the checking interval, and a defect may therefore be unnoticed for a long time. Finally, intermittent failures may not be detected at all by the described methods.

The purpose of the present study was to address the limitations described above by testing an idea of and describing the concept of a novel method of detecting defects related to horizontal nonuniformities in ultrasound equipment. The method is based on the analysis of ultrasound images collected directly from the clinical workflow and applicable to, e.g., linear array transducers, as used in the present study.

## 2 MATERIALS AND METHODS

### 2.A Description of the concept of the method

The proposed method is based on the fact that the anatomical information in an image varies for every clinical image, whereas the darker region corresponding to a defective transducer is present in all images produced by the transducer. Thus, by averaging many images or determining their median, a more or less homogeneous background is obtained even from clinical images, since the anatomical variations tend to cancel each other, whereas the systematic darker streaks — present in all images — remain and become easier to detect than in a single image.

**Figure 1:**
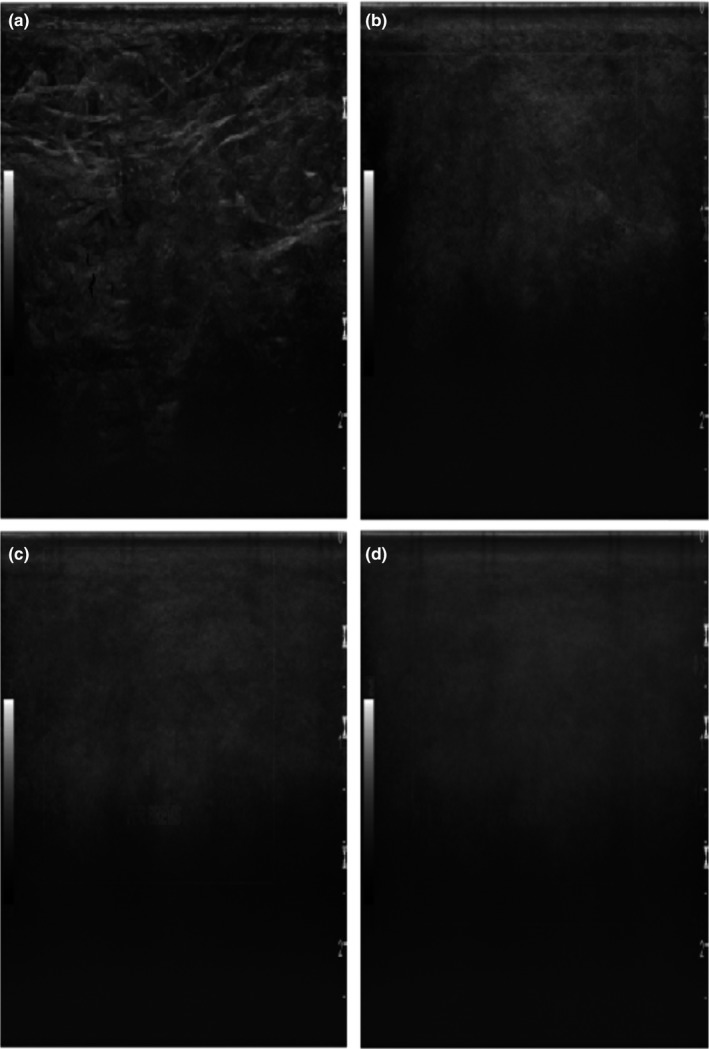
A median uniformity image based on 5, 15, 30, or 100 clinical images (for a, b, c, and d, respectively) produced by a linear array transducer (Case 1). Eight elements were defective according to a FirstCall measurement. An example of a single clinical image acquired with the defective transducer is shown in Fig. 2.

**Figure 2:**
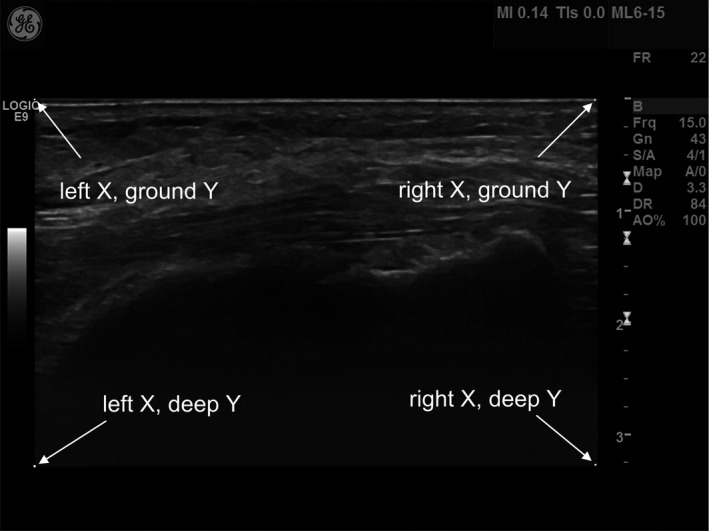
The location of the outer coordinates of the image that was extracted from the original image.

Basing a method of detecting nonuniformities on clinical images introduces new possibilities for quality control. As the images can be collected directly after they are stored clinically, the quality control can be performed without intervening with the equipment and at any time point. The most straightforward approach seems to be to calculate a median uniformity image for visual assessment based on a number of images that have been stored in a database. In Fig. 1, this is shown for different numbers of images where a transducer with eight defective elements (as determined by a FirstCall measurement) was used (Case 1, see Table 1). An example of a single clinical image acquired with the defective transducer is shown in Fig. 2, indicating that it may be very difficult to detect the defects in the clinical images directly.

**Table 1:** The three different cases used for the automated analysis

	Case 1	Case 2	Case 3
Scanner	GE Logiq 9E	GE Loqiq 9E	Philips IU22
Transducer	ML 6‐15	ML 6‐15	L12‐5
Number of images collected	11947	9128	17266
Number of images rejected due to Doppler curves	13	60	1906
Number of images rejected due to outside transducer width limits (over/under)	762 (2/760)	256 (17/239)	3935 (658/3277)
Number of images used	11172	8812	11425
Analyzed period	30 months, 7 days	25 months, 10 days	55 months, 7 days
Image size	720 × 960	720 × 960	768 × 1024

For continuous monitoring of the system, a more advanced approach could be to automatically analyze the latest produced images for horizontal uniformity aberrations. Such an analysis could be a complement to the normal quality assurance program and notify the service organization of a detected artifact and thus lead to earlier detection of defects. The details of an example of such an analysis are given in the present paper. The purpose of the paper was to describe the details of this first attempt in a case study based on three ultrasound systems and to test if it may be possible at all. The results are qualitative and a follow‐up study is planned for a quantitative evaluation of a larger number of ultrasound systems and cases.

### 2.B Application of the method to three general imaging ultrasound scanners

The method for visual assessment and an implementation of an automated analysis was applied to stored images for three scanners, with one defective transducer each. Four FirstCall measurements, revealing different kinds of defects, were available for comparison. The scanners were used in radiological departments for general imaging and the images were stored as 8‐bits RGB (Red Green Blue) images in DICOM (Digital Imaging and Communications in Medicine) format. The scanners, the transducer types and the number of images used retrospectively for the automated analysis are shown in Table 1.

This retrospective study using clinical images was approved by the Regional Ethical Review Board. All image handling and analysis were performed automatically using in‐house developed MATLAB (MathWorks, Inc., Natick, MA, USA) applications. Images from a chosen scanner/transducer combination were imported and selected. The B‐mode part in the stored images was extracted from the surrounding information and was resized so that all extracted B‐mode images had the same size. The extracted and resized B‐mode images were then placed in an image stack, used as input to an algorithm developed for automatic detection of defects. The detection was based on construction of a curve representing systematic darker streaks in the images.

### 2.C Image selection

Images from a certain scanner, distinguished with the DICOM tag “StationName”, were retrieved from the archive. The transducer type was determined by the DICOM tag “TransducerData” for the Philips scanner. For the GE scanners, the transducer information in the images was used instead since the DICOM tag was empty for these images. Images containing Doppler curves and side‐by‐side images were rejected. This was determined by checking if the DICOM tag “SequenceOfUltrasoundRegions” had more than one item or if the size of the image had more columns than the ones just containing one image. Color Doppler images without curves were included.

### 2.D. Image extraction

The stored images had surrounding information around the B‐mode image — fields containing patient name, time, etc. — and the only information needed was the grayscale B‐mode image. To extract the B‐mode image from the surrounding information, the image was first converted from color to gray. The largest connected area of nonzero pixels was then selected using the MATLAB's Image Processing Toolbox functions “bwconncomp” and “regionprops”. The rest of the pixels in the image were set to zero. The coordinates for the outer corners of the selected area were then determined (see Fig. 2) and the B‐mode image was extracted. The determination of the coordinates was based on separating fields of nonzero pixels from zero‐filled fields. Virtually convex images were extracted as a rectangle with the width of the most superficial part the image, and the height equal to the depth of the B‐mode image.

To check if the image was zoomed laterally, the physical aperture of the transducer was compared with the physical width of the B‐mode image, using the left and right X values and the DICOM tag “PhysicalDeltaX”. If the physical width of the B‐mode image was inside the range of 49.5 and 51.5 mm, the B‐mode image was extracted, otherwise the image was rejected (the same range was used for both transducers; the physical aperture was 50 mm for both types). Finally, the extracted B‐mode images were resized in order to get the same size for all extracted images using the MATLAB's Image Processing Toolbox function “imresize” (bicubic interpolation). The width of the resized images was chosen to be equal to the number of elements in the transducer. The height of the resized images was chosen to be 500 pixels for both transducers in order to get decent resolution of the depth. This resulted in image sizes of 336 × 500 for ML 6‐15 and 256 × 500 for L12‐5.

### 2.E Implementation of an algorithm for automatic detection of horizontal nonuniformities

The idea of the automatic analysis was to develop an algorithm that uses the information in the superficial part of a large number of images to create a curve with a length equal to the number of columns in the resized images (and also number of elements in the transducer). When the algorithm detects darker regions above chosen thresholds, these positions in the curve are replaced by positive values that represent the dark regions in width and strength. When the area under this systematic dark region (SDR) curve reaches a chosen threshold, this could be used for a notification that the transducer or the scanner needs an extra check. A 3D plot of the SDR curves could be used to monitor the condition of a system and see if an accepted aberration increases or remains the same.

**Figure 3:**
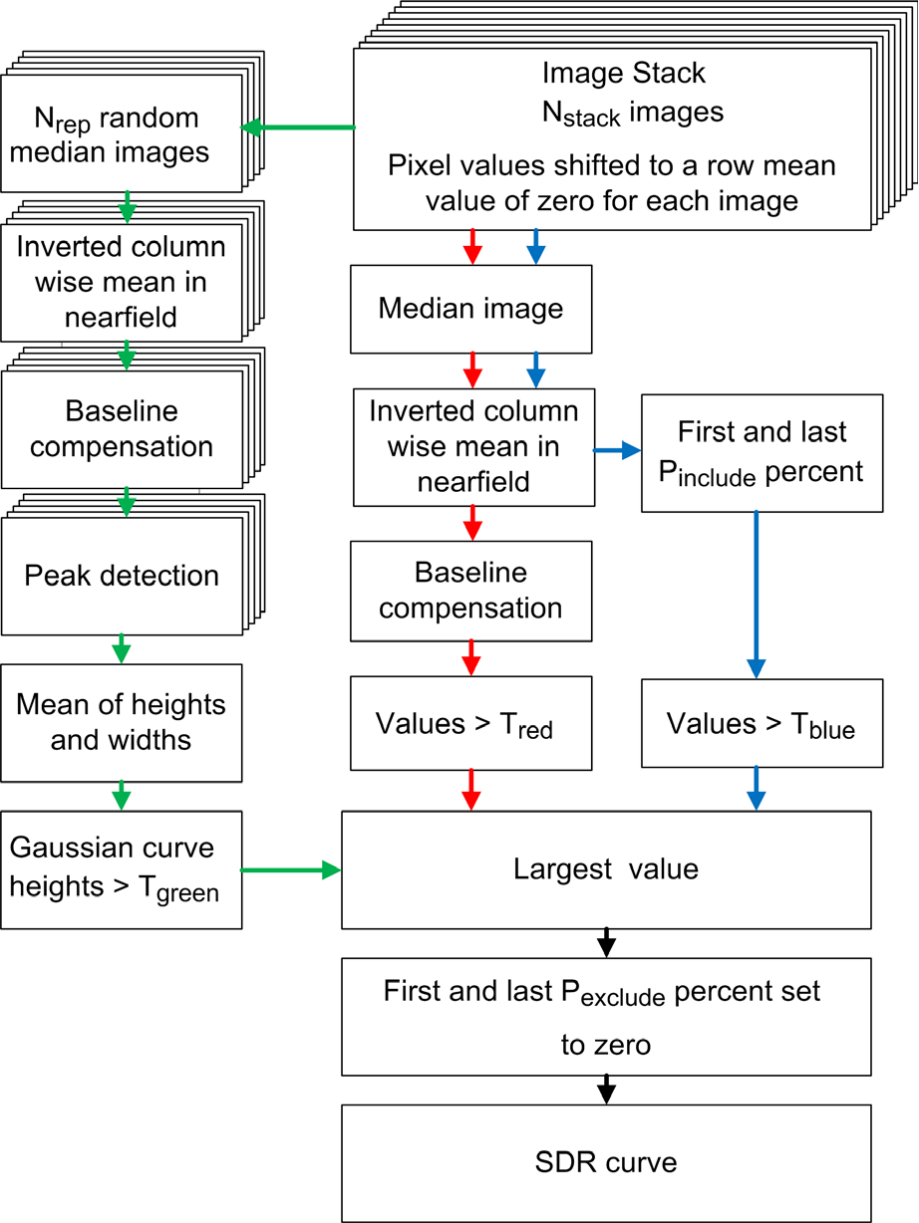
Description of the three paths used by the algorithm to create an SDR curve from an initial stack of N_stack_ images.

In Fig. 3, the different steps of this initial algorithm, used for construction of one SDR curve based on the information in an image stack of N_stack_ images, are shown. The algorithm combines three different types of analyses, described by the green, red, and blue paths in the figure. Each of the three paths creates a curve with a length equal to the number of columns in the resized images and all the values are set to zero as default. The values are changed only if dark regions are determined as aberrations by the algorithm. The largest value of the three curves at each position is chosen for the SDR curve. In the green path, dark streaks that are narrow and situated in the same lateral position in many images are detected. The red path detects wider darker streaks that are more diffuse and not necessarily have the same lateral position of the dark valleys in the images in the stack. Both the green and red paths use subtraction of a polynomial fit to compensate for baseline variations. This approach is not successful if the darker regions appear in the horizontal endpoints of the images since the polynomial fit adopts to the endpoints of the curves. The blue path has the sole purpose to detect darker regions in the horizontal endpoints of the images. The algorithm was implemented in the following way.

First, the mean of each row was subtracted for each row in all images. For the green path, a median image was calculated based on N_select_ randomly chosen images (without replacement) from the image stack. This was repeated N_rep_ times, resulting in N_rep_ median images. A column‐wise‐mean (CWM) of rows R_upper_ – R_lower_ was calculated for each of the median images, resulting in N_rep_ CWM curves. The curves were inverted and a polynomial fit of order O_poly,green_ was fitted to and subtracted from each of the curves. A peak detection was performed using the MATLAB's Signal Processing Toolbox function “findpeaks” (default settings) for each of the curves. The function returned the position, height, and width of all peaks that were found. The means of the heights and widths of the peaks were calculated. All peaks that had a higher mean value of the height than the threshold T_green_ were selected.

The heights and the widths of the n selected peaks were used to create a vector, containing Gaussian curves for the selected peaks at the correct position, see eq. (1).

(1) $$f\left(x\right)=\sum_{k=1}^{n}height\left(k\right)e^{-{\left(\left(x-pos\left(k\right)\right)/width\left(k\right)/2\right)}^{2}}$$

For the red and blue paths in Fig. 3, all N_stack_ images were used to calculate a median image. Pixels between rows R_upper_ and R_lower_ were used to calculate the CWM curve, as above. The CWM curve was inverted and for the blue path all values above T_blue_ for the first and last P_include_ percent of the values in the inverted CWM curve were selected, the rest were set to zero. For the red path, a polynomial fit of order O_poly,red_ was fitted to and subtracted from the curve as baseline compensation. All values above T_red_ were selected, the rest were set to zero.

**Figure 4:**
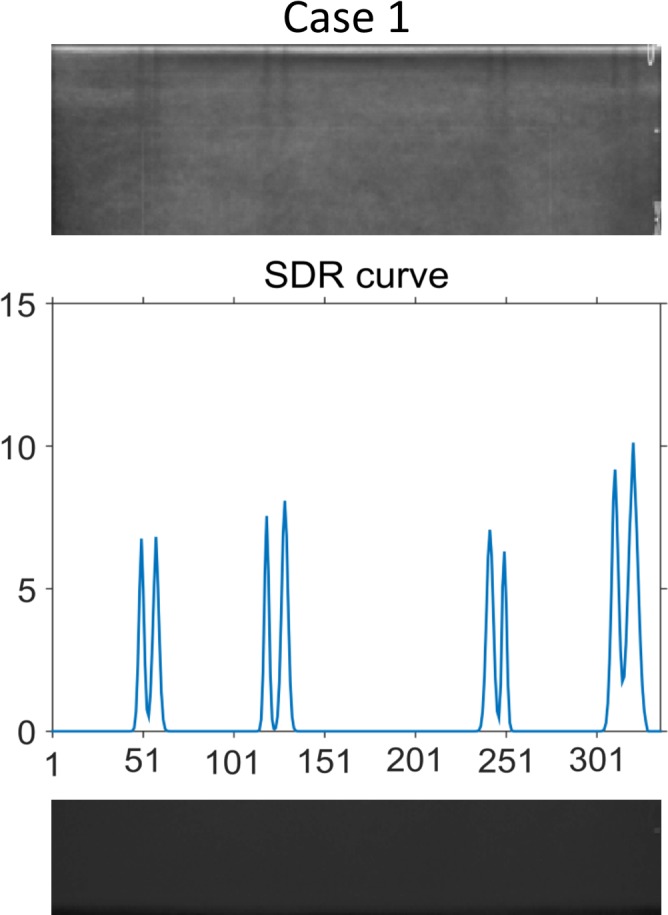
An example of an SDR curve for Case 1, representing the darker streaks in the median image in the background.

At each position, the largest value from the three curves was selected to create the SDR curve. To avoid the darker streaks in the borders of the images that appear for fully functional transducers, the first and last P_exclude_ percent of the SDR curve were set to zero. In order to obtain a scalar measure of the nonuniformity of the system, the area under the SDR curve was finally calculated using trapezoidal numerical integration. The area under the curve was normalized by the number of elements in the transducer. An example of an SDR curve is shown in Fig. 4, overlaid on a median image of the 150 images (N_stack_ = 150) selected from Case 1 for creating the SDR curve.

The algorithm was applied to the image data described in Table 1. All images for each scanner/transducer combination were sorted by the DICOM tag “StudyDate”. The image stack that was used for calculating the SDR curve was updated with a single image at a time. In all, 30962 SDR curves were determined, each representing the status of one of the systems at a given time point.

The values of the variables used were N_stack_ = 150, N_select_ = 15, N_rep_ = 100, R_upper_ = 1, R_lower_ = 19, O_poly,green_ = 6, O_poly,red_ = 6, T_green_ = 2, T_red_ = 5, T_blue_ = 10, P_include_ = 25, and P_exclude_ = 2. These values were selected after empirical testing with the aim to get a result that agreed reasonably well with the FirstCall measurements for the three cases and for other scanners that were investigated but not used in the present paper, but a proper optimization of the parameters was not conducted. The depth distal to the transducer using these values was 19/500 (approximately 4%) of the extracted image depth, regardless of the depth of the image.

## 3 RESULTS

The amount of extracted and rejected images of the image extraction subroutine for the different cases is presented in Table 1. For the two GE cases, the extraction was successful in most cases, whereas in the Philips case, the fail rate was higher as a result of a logotype being present and sometimes disturbing the image.

**Figure 5:**
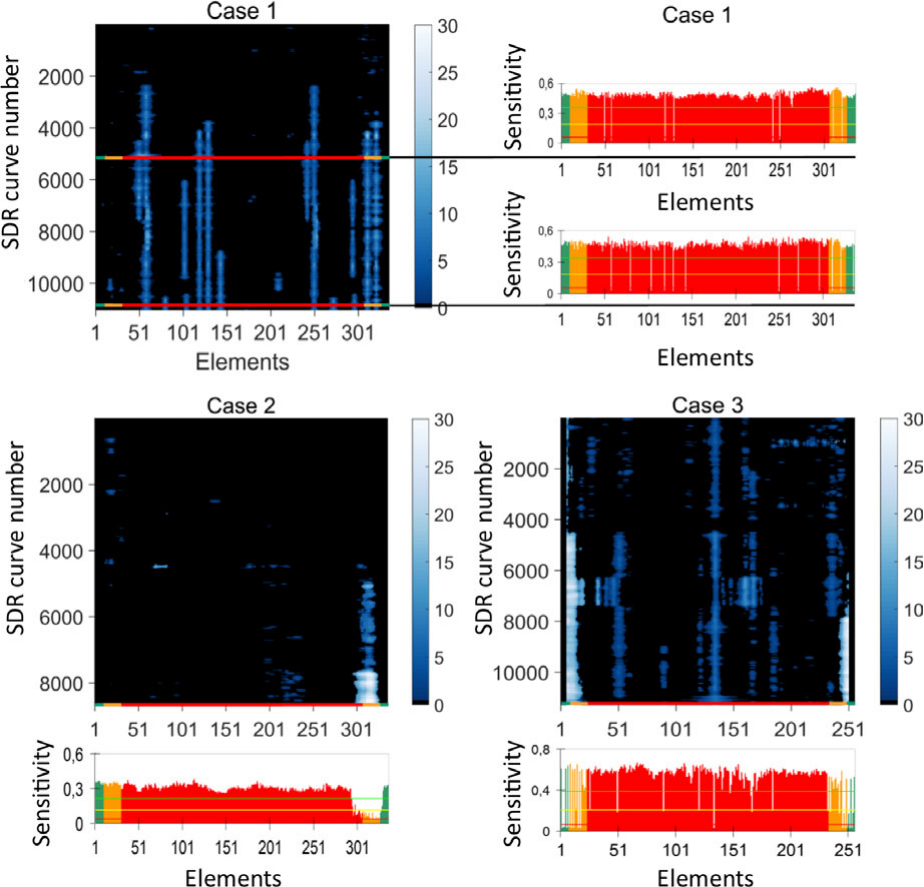
3D plots of the SDR curves for Cases 1–3 and the reports (element sensitivity) from four FirstCall measurements performed on the three transducers. The time interval between the first and last SDR curve was approximately 30, 25, and 54 months for Cases 1, 2, and 3, respectively. The four SDR curves corresponding in time to the four FirstCall measurements are marked (multicolored lines).

The retrospective SDR curves for the three cases are shown as 3D plots in Fig. 5. The SDR curves corresponding to the oldest images have the lowest SDR curve numbers. For all three cases, the algorithm detected several nonhomogeneities, some indicating reduced transducer element sensitivity (sharp peaks in the SDR curve, corresponding to defects in single elements) and others indicating that several elements were affected (broad dark regions). Subjectively, the algorithm seemed to be robust, as visually similar SDR curves were obtained for images acquired at different time points over long periods of time.

For Case 1, there were two FirstCall measurements, the second one performed 16 months after the first one (see Fig. 5). Two of the elements that were reported broken (50 and 242) in the first measurement were reported functional in the second measurement, while new faulty elements were detected. By studying the SDR curves for Case 1 in Fig. 5, it is possible to see approximately when element 50 and 242 became functional after being defective and when the new faults emerged, except for element 335, the location of which was cut away by the last P_exclude_ percent in the algorithm.

For Case 2, it is possible to follow how the defect emerged in both width and strength in Fig. 5. The time period between the first indication of a defect and when the transducer was replaced due to the FirstCall measurement was 10 months. The behavior of the defect indicates that it could be due to delamination.

For Case 3 in Fig. 5, there is a pattern in the SDR curves around curves 6300–7400 that cannot be seen in the FirstCall measurement. By visual inspection of the median uniformity image for this period the pattern was found also in the median image, which makes it reasonable to assume that this was an intermittent defect that was present during this period of approximately 1100 images (5 months) for this particular transducer/scanner combination.

**Figure 6:**
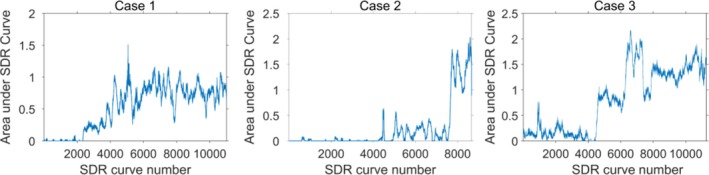
The area under the SDR curve for each SDR curve for the three cases.

The area under the SDR curves for the three cases are shown in Fig. 6. This scalar measure of the horizontal nonuniformity could be used as an indicator for when a check of the transducer (or scanner) is necessary. A comparison between Figs. 5 and 6 indicates that a notification level in the interval between 0.5 and 1 would have detected the defects at an early stage in these cases.

## 4 DISCUSSION

In this paper, a novel method for assessment of the horizontal uniformity of ultrasound systems, based on the use of a large number of clinical images, has been presented. An initial algorithm for automatic detection of systematic vertical dark regions in the superficial part of the images produced by linear array transducers has also been described. The algorithm was applied to three different cases from two vendors/transducers, where four FirstCall measurements were available for comparison. In total 31409 images were used in the analysis by the algorithm, 150 at a time. By visual assessment of the 3D plot of the SDR curves in Fig. 5, the SDR curves showed good subjective visual agreement with the FirstCall measurements; single missing elements as well as larger defects were detected by the algorithm. The method thus seems to be able to provide information about the status of the transducer based on clinical images alone and without the need to actually perform uniformity measurements on the scanner. However, it should be emphasized that the method has not yet been properly optimized or validated. Other ways of extracting the images and algorithms for analysis are possible. Different variants of extracting and resizing the images for other types of 1‐D sequential array transducers could also enable the use of the method for, e.g., curved array transducers. However, a limitation of the present study is that this has not been tested.

The algorithm used in the present paper has similarities to the one described in IEC Technical Specification.^24^ In the IEC method, a column‐wise median curve from the superficial part of a median image, based on 100 images or more acquired of a phantom as a cine loop, is used for quantifying vertical darker streaks. Clinical images have a more complex content than images from a phantom with a homogenous structure. The variations in clinical images are larger and therefore the algorithm used in the present paper was developed; a column‐wise median curve in itself varied too much to be used for the clinical images in the used cases. Therefore, the levels of the SDR curves in the presented method cannot be compared directly with the levels of the curve in the IEC Technical Specification. Furthermore, when a phantom measurement is carried out, settings like dynamic range, spatial compounding and image depth can be controlled and criteria for when to replace or repair the transducer or scanner are the same from time to time. When using clinical images, as in the present paper, all these settings can be different for the images, affecting the magnitude of the deviation in gray level caused by defects in the transducer or scanner. Thus, the area under the SDR curve is only intended to be an indicator of the necessity of a controlled checkup. Nevertheless, the SDR curves may contribute to the overall assessment in quality control. Important advantages are that the evaluation is made in the same way every time and that the images are produced with the settings actually used in the clinic.

The number of images used in the image stack for the construction of each SDR curve, N_stack_, was set to 150 images in the present paper. The higher N_stack_, the more robust the algorithm becomes for systematic defects that are present in all N_stack_ images, but the longer it takes to replace the images in the stack and thus the longer it may take for a new defect to be detected. N_stack_ was set to 150 in order to get illustrative SDR curves for the cases with few false positives, but this number may be reduced for a good balance between occasional false positives and an early detection of defects. To detect intermittent defects it is also preferable to have a smaller image stack. However, a single measurement of a phantom or a FirstCall measurement may not detect intermittent defects. Sipilä et al.^14^ reported that in 11 of 35 tested transducers, occasional dead elements had disappeared when making a yearly follow‐up on transducers using FirstCall. They state that one possible reason for the changes in the results could be occasional bad connections between the FirstCall adapter and the transducer. This is the same type of connector that is used in the scanner, which means that this type of error can be intermittent when the transducer is connected to the scanner as well. Breaks in the cable were the second most reported defect (after delamination) by Mårtensson et al.^12^ Cable failures are by nature typical to sometimes be intermittent. By using the information in the clinical images, the actual defects that are present in the major part of the images are evaluated instead of being dependent on the binary state of an intermittent defect when doing a single measurement. For Case 1, it is possible to follow whether the defects were present or not for individual elements by looking at the SDR curves (Fig. 5). Noticeable for the SDR curves for Case 1 is also that the peaks appear pairwise in the left and right part of the image. This probably depends on cables or connection defects, since this transducer (GE ML6‐15) uses the same cable for two elements for most of the elements.

Since the time from when a defect occurs to when it is discovered should be as short as possible, Mårtensson et al.^13^ concluded that annual testing of the transducers is not sufficient and Hangiandreou et al.^15^ recommended quarterly assessment of both mechanical integrity and visual assessment of the uniformity. A quick scan test (including uniformity test) plus a physical and mechanical inspection is recommended to be performed every 3 months for mobile and emergency room systems and every 6 months for others in the report of AAPM Ultrasound Task Group No.1.^3^ Monthly quick checks that include methods for detection of defective transducers are recommended by EFSUMB Technical Quality Assurance Group^25^ and IEC TS 62736.^24^ The method of automatic detection proposed in the present paper is intended to be a complement to normal quality assurance by continuous monitoring. It could be of use for scanners that are storing enough images for the area under the SDR curve to be affected earlier than the defect would be detected at the normal scheduled quality control or by an electrical measurement of the transducer, like FirstCall. In Cases 1–3, the average clinical image acquisition time period needed to replace the whole image stack when N_stack_ = 150 as used in the present paper, and thus the average number of days for a defect to fully affect the SDR curve, was approximately 12, 13, and 22 days, respectively. The investigated transducers were used in 46%, 14%, and 25% of the images produced by each system for Cases 1–3, respectively. The proportion of images in the image stack containing the defect that is needed to affect the SDR curve depends on the defect characteristics. This has not been investigated, but naturally affects the time for detection.

Using the area under the SDR curve is a simple way to implement an automatic detection of defects. More advanced ways could involve applying weight functions that amplify the central part of the SDR curve more than the peripheral part or that emphasize wider streaks more than narrow ones, etc. In this way, the clinical relevance of the scalar measure might be increased.

The described method has been developed for stored images. One condition for this approach to be successful is that a given transducer is always connected to the same scanner when used, since there normally is no individual information (such as e.g., serial number) in the DICOM tags regarding the transducer; just the type for the Philips machine and none for the GE machines. In the used cases the images were retrospectively collected from a database and there was no possibility to be certain that the same individual transducers had been used for all images. However, the pattern in the SDR curves makes it reasonable to assume that this was the case, since the dark regions appeared in the same lateral positions over time. Generally, if the transducer often is shifted between systems, the method is not suitable. However, if a transducer is defective and connected to another system that is also monitored, the defect will eventually be detected in the new system. Similarly, if the defect remains when transducers are switched, the defect can be assumed to be internal and maybe be tied to a specific port. In this way, the method can be a supplement if the defects are intermittent or if it is difficult to localize the origin of the defect. However, if the intermittent defects occur during periods of time short relative to the total time to fill the image stack, the present method may have difficulties detecting these defects.

An issue when extracting the images was that logotypes and scales sometimes partly interfered on the largest connected region of nonzero pixels. This was handled by detecting the vertical borders in different ways for the two examined manufacturers, and would probably have to be customized for other manufacturers. The used method extracted the whole image and resized both depth and width to get the same number of pixels for both width and depth for all extracted images. This approach makes aberrations in vertical bands not detectable, since different depths are mixed in the median uniformity image. Logotypes and markers set by the users also affected the outcome of the method, since all logotypes and markers are part of the images. A result of this disadvantage can be seen for Case 3 in Fig. 5 for the SDR curves around 1000, in the right part of the curves. The darker detections were caused by distance measurements in many adjacent images, where the results were shown as white text in a black box in the superficial part of the images.

Darker streaks in the image caused by a defective transducer are most easily detected in the superficial part of the image. If an image is zoomed axially, the darker region caused by the defect of the transducer will decrease with depth, but if the image is zoomed laterally, the defect of the transducer and the dark region in the image unfortunately no longer correlate laterally. Therefore, laterally zoomed images were rejected. This exclusion criteria also rejected images if the X values were estimated erroneously by the extraction algorithm.

All scanner settings that affect beam forming, image processing, and possible algorithms that the manufacturers have built into the scanner to compensate for defects in the equipment, affect the clinical image and thus the median uniformity image. If spatial compounding is used, this will probably impact performance of the method to be less sensitive in a similar way as spatial compounding impacts visibility of defects when checking horizontal uniformity using a phantom. For the images used in the present paper, there was no information available whether spatial compounding was used or not. Defects that can be detected using clinical images are also present in the final processed clinical images, which is an advantage if the only purpose is to detect aberrations in the images. However, the major advantages of using stored clinical images are that this can be done automatically and that there is no need to get admission to the equipment — only access to the database where the images are stored is required. If some kind of curves are presented, like the ones in Fig. 5 in the present paper, it is easy to see the location, size and width of the defects and to follow if they are intermittent, steady or increasing.

Assessment of the image uniformity as part of a quality assurance program is used for other imaging modalities as well, such as e.g., computed^26^, ^27^ and digital^28^ radiography systems and in nuclear medicine.^29^, ^30^ To use median images of clinical images for detecting nonuniformities in these systems may also be possible, and could be useful as a complement to quality assurance for early detection of a subset of defects. For systems where the examined part of the patient does not cover the whole detector, or where the examined part like bones or lungs mostly covers the same part of the detector, the median image becomes nonuniform in itself. For such systems, a solution could be to subtract a reference median image of clinical images produced when the detector is known to be fault free.

Finally, to use the information available in clinical images for automated detection of changes in important parameters has been described for other areas and purposes in the field of medical imaging. Examples are automated detection of changes in patient exposure^31^, ^32^ and image quality^33^, ^34^, ^35^ in radiography and computed tomography. Such methods are in line with the Medical Physics 2.0 initiative,^36^ emphasizing, e.g., the necessity for clinical imaging physics to focus on efficient methods and performing clinically relevant tasks. The method introduced in the present work can hopefully contribute to a more effective use of the ultrasound equipment by providing early detection of transducer defects, in the end thus potentially leading to an increased clinical performance.

## 5 CONCLUSIONS

A method of using clinical images for assessment of horizontal uniformity in ultrasound imaging has been introduced. The method is applicable to, e.g., linear array transducers, as used in the present study. An algorithm for automatic detection of horizontal nonuniformities has been described and tested on more than 31000 clinical images from three systems in a case study. Subjectively, the algorithm seemed to be robust, as visually similar SDR curves were obtained for images acquired at different time points over long periods of time. Furthermore, the results showed good visual agreement with available electrical measurements of the defective transducers, indicating a potential use of clinical images for early and automatic detection of defective transducers, as a complement to quality assurance.

## CONFLICT OF INTEREST

The authors declare no conflict of interest.
